# Management of patients with liver diseases on the waiting list for transplantation: a major impact to the success of liver transplantation

**DOI:** 10.1186/s12916-018-1110-y

**Published:** 2018-08-01

**Authors:** Didier Samuel, Audrey Coilly

**Affiliations:** Centre Hépatobiliaire, Paul Brousse Hospital, University Paris South, Inserm-Paris South Research Unit 1193, 12 Avenue Paul-Vaillant Couturier, Villejuif, 94800 Paris, France

**Keywords:** Liver transplantation, Meld score, Hepatocellular carcinoma, Viral hepatitis, Acute on chronic liver failure, Cirrhosis, Liver transplant waiting list, Alcoholic cirrhosis

## Abstract

**Background:**

The results of liver transplantation are excellent, with survival rates of over 90 and 80% at 1 and 5 years, respectively. The success of liver transplantation has led to an increase in the indications for liver transplantation. Generally, priorities are given to cirrhotic patients with a high Model for End-Stage Liver Disease (MELD) score on the principle of the sickest first and to patients with hepatocellular carcinoma (HCC) on the principle of priority points according to the size and number of nodules of HCC. These criteria can lead to a ‘competition’ on the waiting list between the above patients and those who are cirrhotic and have an intermediate MELD score or with life-threatening liver diseases not well described by the MELD score. For this latter group of patients, ‘MELD exception’ points can be arbitrarily given.

**Discussion:**

The management of patients on the waiting list is of prime importance to avoid death and drop out from the waiting list as well as to improve post-transplant survival rates. For the more severe cases who may swiftly access liver transplantation, it is essential to rapidly determine whether liver transplantation is indeed indicated, and to organise a fast workup ahead of this. It is also essential to identify the ideal timing for liver transplantation in order to minimise mortality rates. For patients with HCC, a bridge therapy is frequently required to avoid progression of HCC and to maintain patients within the criteria of liver transplantation as well as to reduce the risk of post-transplant recurrence of HCC. For patients with cirrhosis and intermediate MELD score, waiting time can exceed 1 year; therefore, regular follow-up and management are essential to maintain the patient alive on the waiting list and to achieve a good survival after liver transplantation.

**Conclusion:**

There is a diversity of patients on the waiting list for transplantation and equity should be preserved between those with cirrhosis of high and intermediate severity and those with HCC. The management of patients on the waiting list is an essential component of the success of liver transplantation.

## Background

Liver transplantation plays a major role in the therapeutic path of liver diseases [1]. It concerns all patients with end-stage liver disease when other medical therapies have failed as well as a high number of patients with primary liver cancer, mainly hepatocellular carcinoma (HCC). The success of liver transplantation over the past years has been considerable, with more than 5000 being performed annually in Europe, surmounting to more than 140,000 liver transplantations in total [2]. Such success comes as a consequence of the absence of alternative therapies and of the good post-transplant survival of approximately 90 and 80% at 1 and 5 years, respectively.

In Europe, liver donors are mainly of cadaver origin, either following brain death or cardiac arrest, under the Maastricht 3 procedure [1]. In contrast to living donor transplantation, where the date of liver transplantation is known in advance, in cadaveric donor liver transplantation the date of liver transplantation is unknown. Therefore, the wait for transplantation can vary from a few days in the case of acute liver failure patients to more than 1 year for patients with cirrhosis of intermediate severity or those with HCC. The waiting lists for liver transplantation are composed of three types of patient. (1) Those with acute liver failure, included, in most countries, in a super-emergency waiting list giving them absolute priority over all other recipients, and receiving a transplant within hours or days [3]. (2) Those with decompensated liver cirrhosis, included in the waiting list on the principle of the ‘sickest first’, based mainly on the calculation of the Model for End Stage Liver Diseases (MELD) score. Those with very high MELD score may likely access liver transplantation within days or a few weeks, whereas those with intermediate or low MELD scores will access liver transplantation within months or even years. (3) Those with HCC on compensated liver cirrhosis. Organ-sharing agencies assign these patients an artificial score according to the number and size of tumours, providing them access to liver transplantation within a maximum of 18 months. Since the number of candidates on the waiting list is greater than the offer of viable livers, there is some degree of competition between patients. Liver centres should manage their patients according to their clinical situation and the expected waiting time for liver transplantation. For a patient with an end-stage liver disease, the procedure of liver transplantation and the postoperative period remain a heavy procedure. Therefore, patients should be carefully prepared physically and mentally to undergo transplantation, much like an athlete preparing for the Olympic games. The management of patients on the waiting list is essential to avoid death or drop out due to deterioration of their condition as well as to ensure that patients are in the best physical condition possible prior to the procedure – this is essential for the success of the post-operative course.

### Management of patients with decompensated liver cirrhosis

In the USA, the MELD score has been implemented since 2002 as a score to prioritise access to liver transplantation [4]. Most countries in Europe have implemented the principle of the ‘sickest first’ to prioritise such access also based on the MELD score, despite some differences between countries. The principle of the ‘sickest first’ is one of justice and has achieved a reduction in mortality whilst on the waiting list in most countries. However, there are several inherent issues that must be addressed.

First, the MELD score, calculated from bilirubin, creatinine and international normalised ratio levels, is not the holy grail [4–6]. Some patients with severe liver diseases are poorly represented by the MELD score, such as, for example, patients with cholestatic diseases, who have, until the very late stage, a low MELD score due to normal international normalised ratio and creatinine levels, patients with refractory ascites and otherwise preserved liver function, patients with chronic encephalopathy due to portacaval shunts, and patients with hepatopulmonary syndrome. Thus, these patients should be offered MELD exception points to provide them the possibility to access liver transplantation [7].

### Management of patients with high MELD score or in the intensive care unit (ICU)

There is a debate regarding the high mortality risk after liver transplantation in patients with a high MELD score [8]. Some authors consider that transplantation in these patients can be futile. On the one hand, the survival benefit for these patients is extremely high as shown in patients with severe acute alcoholic hepatitis or with acute-on-chronic liver failure [9, 10]; on the other, the high mortality rates after liver transplantation can be considered a waste of organs [8]. The MELD score is highly predictive of the risk of mortality on the waiting list [4, 11]. Patients on the waiting list with a MELD score of over 30 or 40 have an expected mortality rate of more than 50 and 70%, respectively, within 3 months [4]. Therefore, there is justification to prioritise these patients. In patients with very high MELD scores (> 35), there is a risk of high post-transplant mortality and of over indication for liver transplantation. There is a consensus that for scores up to MELD 35, post-transplant survival remains unmodified. In patients with a MELD score above 35, there is currently no consensus on the potentially higher risk of post-transplant morbidity and mortality [12]; however, the postoperative morbidity and ICU length of stay are significantly higher [12, 13]. Interestingly, the percentage of patients with chronic liver disease transplanted while in the ICU or soon after recovery remains low (< 10%) [14]. There are several reasons for this. First, patients in the ICU are frequently not evaluated for liver transplantation either because they are not in a liver ICU or because liver transplantation is considered too risky and futile. Several authors have tried to determine, through scores such as the Frailty scoring system [15] or other scores [16], the limits at which transplantation is futile. Our personal opinion is that this line of futility is permanently evolving.

Considering the dramatic improvement of the results of liver transplantation over the past years, we need to be more reactive regarding the indication of liver transplantation in the most severe patients, i.e. those in the ICU. When a patient is referred in a critical condition, the very first question to address is: is this patient a potential candidate for liver transplantation? For alcoholic cirrhotic patients, or for patients with acute alcoholic hepatitis refractory to medical treatment, this will require an urgent workup, advice from a specialist in alcohol addiction, and a consensus decision from the team [9]. When the decision for liver transplantation is taken, patient prognosis should be assessed using ICU scores rather than the MELD score [17, 18]. An urgent workup to assess comorbidities is also required. The difficult issue is the definition of the optimal window for transplantation in such severe cases. The risk is to perform transplantation in patients without an adequate workup at the worse moment. Therefore, coordination with the ICU specialists in order to determine the appropriate timing for liver transplantation is essential. The optimal transplantation window between several complications is difficult to determine, but essential for its success. This transplantation window may be open during the ICU stay or soon after recovery prior to the advent of the next deteriorating event [19].

### Management of patients with intermediate MELD score

In many countries, patients with a MELD score between 18 and 25 have difficulty obtaining access to liver transplantation. The waiting time for these patients is long and may exceed 1 year, with a risk of acute deterioration of their liver condition. The position of most organ-sharing agencies is that patients will be offered transplantation as soon as the MELD score increases. However, some patients may deteriorate rapidly and die following new gastrointestinal bleeding or a septic shock episodes. The other option, not yet considered by organ-sharing agencies, is to assign these patients additional points in relation to the length of waiting time on the list, taking into account the natural history of liver cirrhosis. We find it logical to provide such patients with the possibility to increase their priority in the waiting list. During this waiting time, patients should be maintained in the best possible condition until transplantation, being followed on a regular basis either by the transplant centre or by the referring centre. For this, several actions have to be taken, as follows: (1) in hepatitis C virus cirrhotic patients, an antiviral treatment with interferon-free direct antiviral agents should be discussed [20–22]; (2) patients should be regularly screened with Doppler ultrasound of the liver for emergence of HCC; (3) prevention of gastrointestinal bleeding by routine endoscopy should be performed; and (4) patients with refractory ascites should be treated either by TIPS or by iterative paracentesis to avoid malnutrition and other complications of refractory ascites. Additionally, it has been shown that physical activity during the waiting time is beneficial for the patient and may improve its VO_2_ [23].

### Management of patients with HCC

Patients with HCC on decompensated liver cirrhosis may have access to transplantation through the MELD score. However, most of the patients with HCC on the waiting list have a compensated liver cirrhosis and a low MELD score. Thus, these patients cannot get access to liver transplantation through the MELD score. Therefore, patients with HCC on the waiting list will gain points according to the size of the tumour and the number of nodules as well as waiting time duration. There are some differences between countries, yet the principle is highly similar. In general, no additional points are given for TNM1 tumours (a single nodule below 2 cm) where strategies other than liver transplantation should be favoured (i.e. percutaneous radiofrequency, surgical resection). In contrast, patients with TNM2 tumours will gain points with waiting time. The time between listing and transplantation depends on the number of points given to these patients and may vary according to the policy of the organ-sharing agencies. During this period, the centres should have a strategy to limit or reduce tumour growth in order to avoid drop out from the waiting list. It has been shown that a bridge therapy for HCC is necessary for projected waiting times of over 6 months. The treatment of HCC during this period may have a double objective; first, to reduce the size and number of active tumours in order to place patients within the criteria for transplantation (downstaging of the tumour) and, second, to reduce and avoid tumour progression. The treatment strategies for HCC may include percutaneous radiofrequency, surgical resection of some tumour nodules, transarterial chemoembolisation and, in some cases, sorafenib [1]. The treatments can be combined according to the type and the progression of HCC. Despite these strategies, some patients will drop out from the list due to the progression of HCC outside the criteria for transplantation. The three main poor prognosis factors in patients with HCC are the regular increase of AFP, an AFP level of over 1000 ng/mL and the invasion of the portal vein by the tumour [24, 25]. These factors can become a contraindication for liver transplantation due to the high risk of recurrence after liver transplantation.

### Is there a competition on the waiting list between patients with decompensated liver cirrhosis, patients with MELD exceptions and patients with HCC?

Since the points assigned to patients with HCC are given arbitrarily, there is a risk of disequilibrium in favour of these patients depending on the weight given to these points. It is true that, in most countries, the percentage of patients on the waiting list with HCC is increasing, and is currently at 30–40%. Such an increase is due to the epidemiology of liver diseases, the increasing number of patients with HCC and the high benefit in survival by liver transplantation to patients with HCC. In addition, the criteria for liver transplantation for HCC have evolved. Since 1996, the Milan criteria (one nodule of ≤ 5 cm or three nodules ≤ 3 cm without vascular invasion) were the validated criteria for liver transplantation for HCC [26]. With these criteria, the recurrence of HCC after liver transplantation was below 10%. The Milan citeria are still the validated international criteria. There is a push from several teams to expand these criteria. In France, the Milan criteria have been replaced by the AFP score [24] . Therefore, several new criteria have been proposed. The UCSF criteria (1 nodule ≤ 6.5 cm or *n* ≤ 3 nodules ≤ 4.5 cm or total sum ≤ 8 cm) [27]; the up to seven criteria (number of nodules + the maximum size of the tumour without vascular invasion should be 7 at maximum) [28]; and the AFP score, taking into account the size, number of nodules and the level of AFP (applied in France) [24].

Due to the improvement in efficacy of antiviral treatments, the number of patients with decompensated, hepatitis B and C virus liver cirrhosis is sharply declining [29]. In contrast, the number of patients with decompensated liver disease due to alcoholic cirrhosis has shown no decreases, and the number of patients with decompensated liver disease due to non-alcoholic steatohepatitis has been increasing, as shown already in the USA [30].

Thus, it is highly important that organ-sharing agencies maintain a good balance between the different indications for liver transplantation, thus avoiding an increase in mortality or drop out from the waiting list for any one category of patients. It is important to provide liver transplantation access to the sickest patients with decompensated liver diseases, as well as to patients with HCC and those with decompensated cirrhosis and intermediate MELD score. Some authors consider that patients with MELD exceptions should be prioritised over those with very high MELD scores. The perfect equation does not exist, yet a real time assessment of the dynamic of the waiting list by the organ-sharing agencies is essential to maintain equity.

## Conclusion

Physicians should indicate liver transplantation for those who will gain the most benefit from it, and should optimise the management of these patients during the waiting period in order to reduce the rate of dropout and to improve the results after liver transplantation. Finally, given the shortage of organs, physicians should balance the benefit for the society with those to individual patients. These should not be in opposition and we should work on increasing the organ pool and access to liver transplantation, as well as on improving the results of liver transplantation.
